# Spatial differences and underlying mechanisms in electronic word of mouth in the foodservice industry: A case of Sanya, China

**DOI:** 10.1371/journal.pone.0303913

**Published:** 2024-05-30

**Authors:** Xinjie Yu, Ke Xu, Biao He, Xiangjing Zeng

**Affiliations:** 1 College of International Tourism and Public Administration, Hainan University, Haikou, China; 2 Hainan Provincial Tourism Research Base, Haikou, China; Federal University of Goias: Universidade Federal de Goias, BRAZIL

## Abstract

Studying the electronic word-of-mouth (eWOM) in the foodservice industry can not only provide guidance for merchants, but also spatially optimize the urban foodservice industry, restaurants’ location selection, and customers’ purchasing decisions. In this study, taking Sanya city as the research object, using big data crawling technology to collect the directory and their attribute information of 2107 restaurants with more than 100 reviews. Kernel density analysis, grid analysis and the geographically weighted regression (GWR) model were applied to reveal the distribution characteristics and influencing factors of eWOM in the foodservice industry in Sanya, China. The main results are as follows. The foodservice industry in Sanya extends along the southern coastline and is characterized by little dispersion and agglomeration at the macro level. The overall eWOM score of the foodservice industry is low. Market popularity, restaurant rating, transportation conditions, and commercial development all have a positive impact on the eWOM of the foodservice industry. Population and price have both positive and negative effects and the public services has a nonsignificant impact on the eWOM. This study not only improves the theoretical understanding of the foodservice industry, but also provides a general reference for its development in other industries and cities.

## Section 1: Introduction

The foodservice industry is an important staple of the urban service sector [1], and thus its development can both promote economic growth and increase employment [1]. With the rapid development of the Internet, e-commerce has gradually replaced traditional business models [2, 3]. As a result, customer reviews and electronic word-of-mouth (eWOM) have become an important input in consumers’ choices and assessments of merchants. On the one hand, information about restaurants on electronic platforms gives consumers more choices, provides more dining flexibility, and expands their range of activities [4]. On the other hand, the comments of other consumers can change consumers’ spatial and temporal dining behaviors, which in turn influences their decision-making behaviors [5, 6] as well as restaurant site selection [7–9].

Establishing the spatial distribution of tourism in cities is one of the research on urban tourism. Spatial distribution refers to the distribution and configuration of ecological or geographic elements in space, where geographic elements of different sizes and shapes are spatially arranged and combined to form a random, agglomerated or uniform distribution pattern [10]. The spatial distribution pattern of the foodservice industry is the spatial distribution characteristics and patterns of catering store-related elements within a certain geographic area, including the type of spatial distribution, spatial distribution density, and spatial distribution equilibrium. The spatial distribution and influencing factors of the foodservice industry have previously been studied from the perspective of tourism, mainly in the context of single foodservice formats such as hotels [11], tourist restaurants [12], fast food chains [13], and high-end restaurants [14]. As the availability of spatial data has increased, scholars have begun to use qualitative methods to study the spatial characteristics and influencing factors of the foodservice industry, which include transportation accessibility [15], economic development [16], population density [16, 17], infrastructure [18, 19], locational factors [20], market demand [21], supplier density [22], commercial land prices [23], size and cost [24], and restaurant type [25].

Furthermore, the application of big data mining technology provides new avenues for research. For example, Point of Interest (POI) and review data collected by crawling the Internet were used to study the spatial patterns characteristics of metropolitan foodservice enterprises [15, 26]. The kernel density estimation, cold hotspot analysis, spatial clustering and other methods have been used in these studies [15, 26]. Quantitative analysis methods such as correlation analysis, geodetector software, and geographically weighted regressions have been used to explain the impacts of various factors on the spatial pattern in the foodservice industry [16, 21, 27]. Recently, the Online To Offline (O2O) model has dramatically changed the spatial distribution of restaurants in China. As a result, the distribution pattern and influencing factors of takeaway restaurants have become a prominent research area in recent years [2, 3, 28].

As a product of traditional Internet word-of-mouth, eWOM is becoming a major source of information about products and services that includes comments, ratings, video recommendations, tweets, pictures, and blog posts [29]. Donthu et al. used the bibliometric analysis and systematic review methods to analyze eWOM. They found that there are four primary topics in the current research: determinants of eWOM, eWOM in the hospitality industry, cognitive aspects of eWOM, and service failure and recovery [30]. Several empirical studies have established the impact of eWOM on consumers’ intention to purchase products or services, such as intention to choose a travel destination [31] and intention to book a hotel [32].

Most attention in the study of eWOM in the foodservice industry has been paid to the relationship between online reviews and purchase decisions [33]. It also relies on geographic methods. For example, Xu et al. took 3645 restaurants in Nanjing to construct an eWOM index system for restaurants and concluded that it followed the central flow theory [4]. He et al. analyzed eWOM in the foodservice industry in Nanchang and found that the distribution of restaurants determined the level of eWOM, which in turn determined the development of the foodservice industry [34]. Additionally, the specific activities of the host city can affect its spatial distribution of restaurants [4, 33–35].

Previous research has shown that individualism/collectivism and uncertainty avoidance affect consumer eWOM [36]. Epecifically, collectivists provide less negative eWOM, have higher satisfaction and givehigher ratings. In contrast, individualists are often more negative, provide lower ratings, deviate from the average or general consensus [37]. There is a negative relationship between uncertainty avoidance and review valence. High uncertainty avoidance cultures also give critical feedback on poor service quality as a way to alleviate their post–purchase cognitive dissonance [38].

While there are studies that explore the spatial pattern of the foodservice industry and its influencing factors based on big data, there are still certain aspects that require further exploration. First, the portrayal of the spatial distribution pattern in the foodservice industry was mostly focused, but what are the underlying mechanisms of its spatial structure is vague [1]. Second, previous studies mainly sourced their data from statistical reviews, surveys, and interviews to analyze service quality and customer satisfaction [39]. However, there is almost no research on combining big data technology to study eWOM in foodservice industry. Third, megacities such as London [27] and Beijing [15] have been studied. However, for some small and medium-sized cities, especially coastal cities, the research on eWOM of foodservice industry is relatively lacking.

Based on the above research gap, this study selected Sanya, China as a case to study its spatial distribution and influencing factors of eWOM in foodservice industry. Sanya, as a popular typical coastal tourist city in recent years, has reached a high domestic level in terms of the speed of development of its catering industry, market prospects, service level and diversity of catering types. However, its eWOM in foodservice industry has not been studied. Therefore, studying its influencing factors and spatial heterogeneity of the eWOM in foodservice industry can promote the improvement of the foodservice eWOM and the reduction of regional differences, and then promote the foodservice industry to develop in the direction of high quality and equalization. In this study, specifically, (1) the spatial distribution characteristics of eWOM in the foodservice industry are presented, (2) the influencing factors and spatial heterogeneity of eWOM the foodservice industry are identified, and (3) specific recommendations for improving eWOM in the foodservice industry are made.

The main contributions of this study can be described as follows. First, multiple data sources were used, thus providing a generalizable research framework. Second, based on review data and using the ArcGIS techniques of spatial kernel density and grid analysis, the spatial distribution characteristics of eWOM in the foodservice industry in Sanya are visualized. Finally, the geographically weighted regression (GWR) model was used to explore the spatial heterogeneity of underlying mechanisms in the foodservice industry’s eWOM.

The remainder of this study is organized as follows. In Section 2, a theoretical framework was constructed. In Section 3, the data sources and research methodology are described, which lays the research foundation. In Section 4, the spatial differences are analyzed, aiming at exploring the spatial distribution characteristics of eWOM in foodservice industry in Sanya. Section 5 explores the influencing factors of eWOM in foodservice industry in Sanya, aiming at further analyzing the potential mechanism. Section 6 concludes the research conclusion and discuss it with the existing literature, so as to deepen the research content.

## Section 2: Theoretical framework

**Fig 1:**
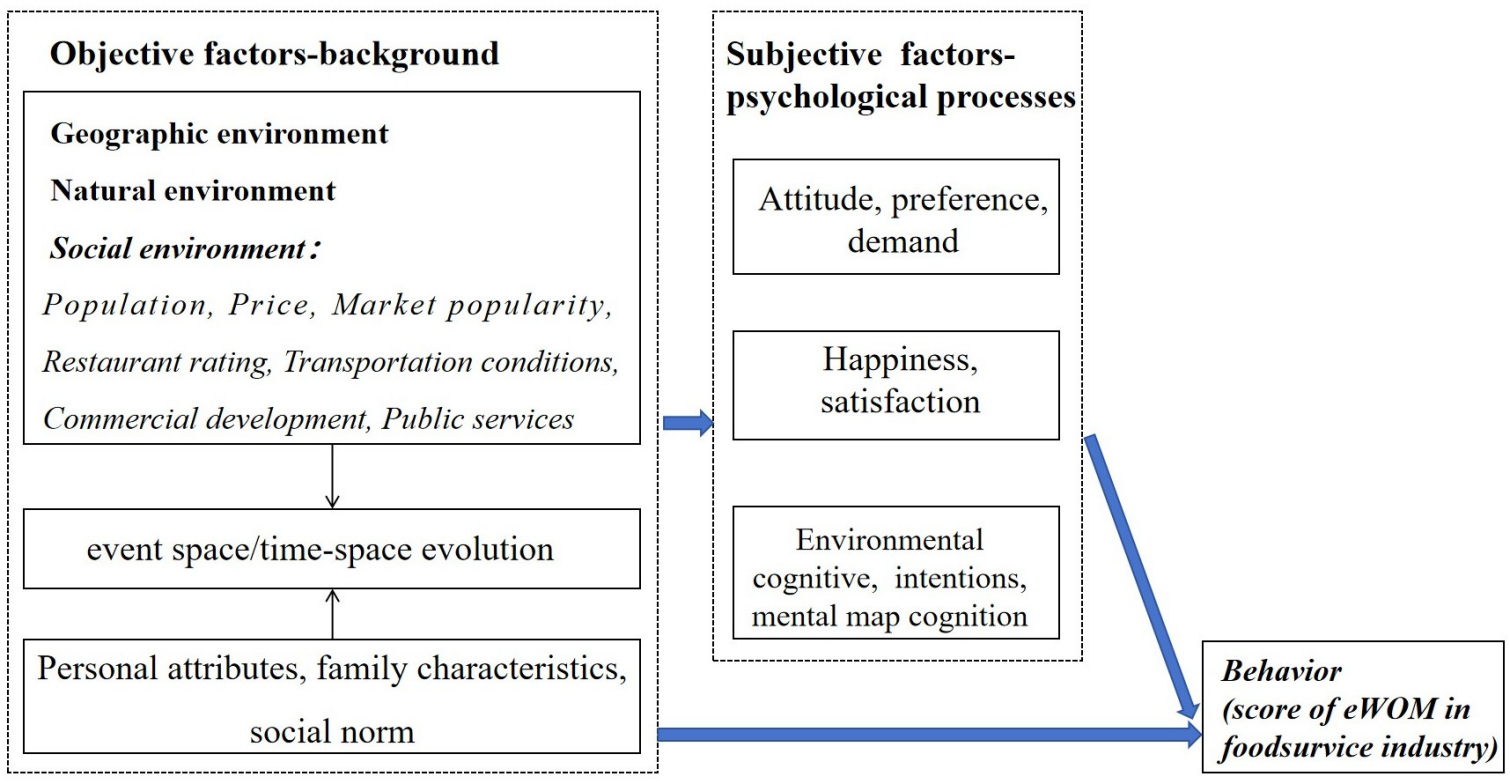
Theoretical framework of this study.

Behavioral geography emerged in the 1960s, which is a branch of geography developed on the basis of psychology, sociology, anthropology, philosophy [40]. Behavioral geography mainly studies the types of psychological behaviors and decisions of human beings in different geographic environments and their formative factors (including geographic, psychological and other factors) [41]. Behavioral geography has become a new methodology of geography research, widely used in human geography and other research fields, such as tourism behavior, consumer behavior, criminal behavior, population mobility, urban life, etc., to a certain extent, expanding and supplementing the research content and methodology of human geography, and promoting the development of human geography [42]. Behavioral geography have constructed an analytical framework of “objective world-cognitive process/emotional-response behavior” [40] (Fig 1).

In this study, geographical methods are mainly used to carry out related research. Therefore, the italicized part in Fig 1 is the key research content of this study, that is, seven factors in Sanya will affect customers’ eWOM of restaurants. Specifically, on the basis of considering data availability, geographical research perspective and existing literature, seven factors are selected as influencing factors, namely: population, price, market activity, food quality, transportation conditions, commercial development, and public services. Therefore, the theoretical framework of this study has been constructed.

## Section 3: Materials and methods

### Study area

**Fig 2:**
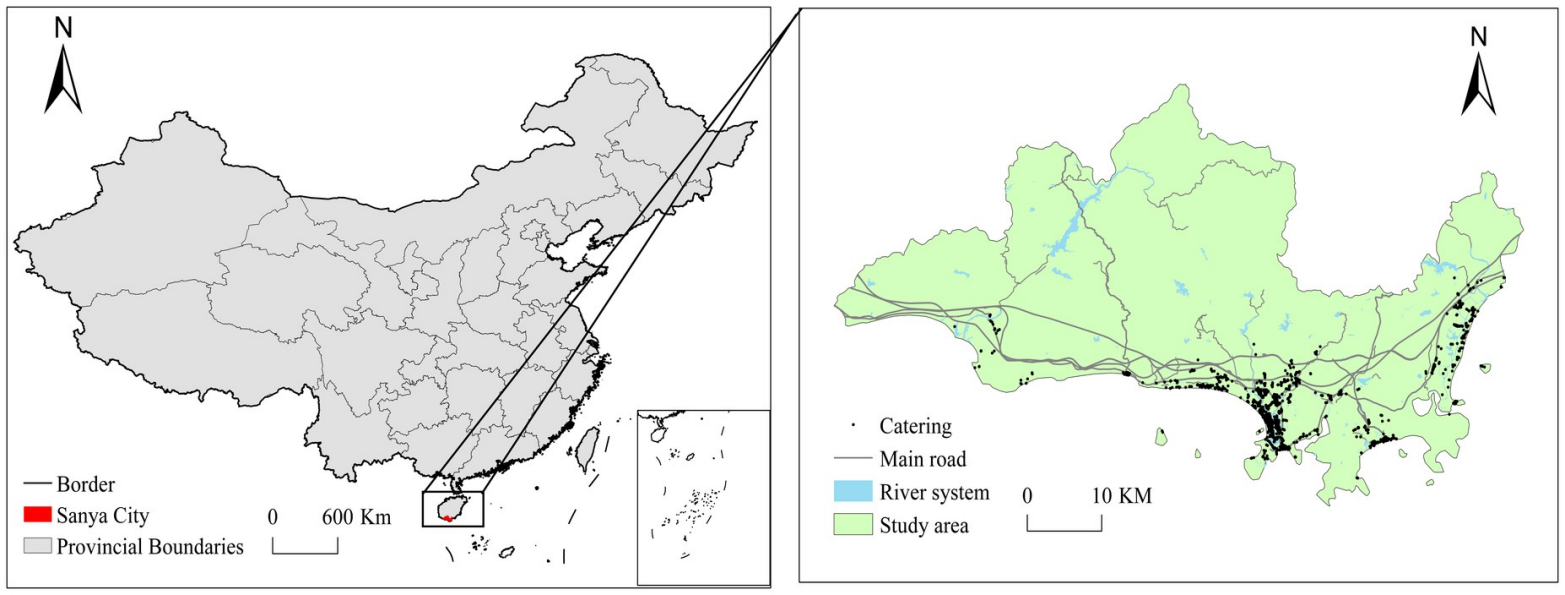
Location of the study area. Note: This drawing is based on the standard map Joan S(2021)024 of Hainan Surveying and Mapping Geographic Information Bureau of the Ministry of Natural Resources, and the boundary has not been modified. Same as the full text.

The area of interest in this study is Sanya in China (Fig 2), which has a total land area of 1919.58 square kilometers, including four municipal districts, namely, Yazhou, Tianya, Jiyang, and Haitang. In 2021, the city received 21,620,400 overnight visitors and the total tourism revenue was 74.703 billion yuan. Some developments such as the construction of the Hainan free trade port and international tourism center have provided opportunities for Sanya’s foodservice industry to grow. This is also an important reason for our selection of Sanya as a case study object.

## Data sources

The review data, which were collected in March 2023, come from the public review website (http://www.dianping.com). Big data crawling technology was used to collect the data for restaurants with over 100 reviews as well as their attribute information (i.e., address, per capita consumption, restaurant type, store rating, food rating, environment rating, service rating). In this public review website, these scores used in this study are all open and free. Then, the data were cleaned, screened, deweighted, and preprocessed, which left us with 2107 restaurants. The geographical information of hotels (i.e., their latitude and longitude coordinates) were queried in batches using Baidu Maps, and then the data were verified through field research and random telephone interviews. In this study, the infrastructural data for Sanya were digitally acquired through remote sensing image alignment. The POI data were sourced from the Gaode Maps platform in April 2023. The data included six major categories: (1) public transport stops, (2) shopping centers, (3) hotels, (4) leisure areas, (5) scenic spots and (6) scientific, educational, and cultural venues. The data used in this study from the Gaode Maps platform is all open and free. In fact, there have been many research results on electronic scoring using POI data [1, 15, 27].

### Research methodology

#### Kernel density analysis

Kernel density can reflect the spatial distribution of point data regularity, and thus it is widely used to reflect the degree of spatial clustering in the point data. It is mainly based on the number of point elements in each unit area and takes different weights to differentiate the resultant smoothness to generate a continuous density surface [43]. In this study, kernel density analysis was used to reflect the spatial distribution characteristics of different types of foodservice industry in Sanya. The formula is as follows:

$$f\left(x\right)=\frac{1}{nh}\sum_{i=1}^{n}k\left(\frac{x-x_{i}}{h}\right),$$ (1)

where *K*(·) is the kernel function and *h* is the broadband. The value of *h* has a significant impact on the results such that the greater the value of *h* is, the smoother the spatial point density variation will be and vice versa.

### Grid analysis

Due to the large differences in municipal districts and streets in Sanya, the administrative division area can not reveal the spatial distribution characteristics of eWOM in foodservice industry well. Therefore, smaller spatial units need to be delineated by creating smaller maps. Grid analysis just meets this requirement.

Grid analysis is one of the effective methods to describe, analyze, and virtualize regional geographic phenomena based on spatial coordinate system and it has a wide range of prospects in spatial pattern analysis. A ‘fishing net’ is a regular and uniform grid. The grid’s incremental unit, the analysis of impact factors can substantially improve the accuracy of the results, as each grid area is equal and comparable. After several debugging iterations using the Create Fishnet tool, 1 km * 1 km grid units were selected and a total of 1824 evaluation units were created after eliminating the irregular edges. After the grid division, 1824 grids were used to extract the mean values of overall restaurant industry ratings and single-dimension ratings in each grid as a characterization of the eWOM in foodservice industry in this unit, and then ArcGIS was used for visualization and observation of the results.

### Geographically weighted regression (GWR) model

The GWR model is an improvement of the homogenization phenomenon of general linear regression, whose regression coefficient β is no longer a uniform single value globally, but βi that varies with the spatial location i. Thus, it can reflect the change of the influence (elasticity) of the explanatory variables on the explained variables with the spatial location. The model explores the spatial changes of the research object at a certain scale and related driving factors by establishing local regression equations of the research variables, which takes into account the local effects of the spatial object [44]. In this paper, the GWR model is used to reveal the spatial heterogeneity of the impact of each influencing factor on the eWOM in foodservice industry. The formula is as follows:

$$y_{i}=\beta_{0}\left(u_{i},v_{i}\right)+\sum_{i}^{n}\beta_{k}\left(u_{i},v_{i}\right)x_{ik}+\varepsilon_{i},$$ (2)

where *y*_*i*_ is the dependent variable, *x*_*ik*_ is the independent variable, *u*_*i*_ is the coefficient on sample *i*, *v*_*i*_ is the value of the continuous function in sample spatial cell *i*, and *ℇ*_*i*_ is the random error term.

## Section 4: Results

### Spatial distribution characteristics of the foodservice industry

Kernel density maps were generated using the ArcGIS10.8 spatial analysis tool to visualize the spatial pattern of the various types of restaurants in Sanya.

### Overall distribution characteristics

**Fig 3:**
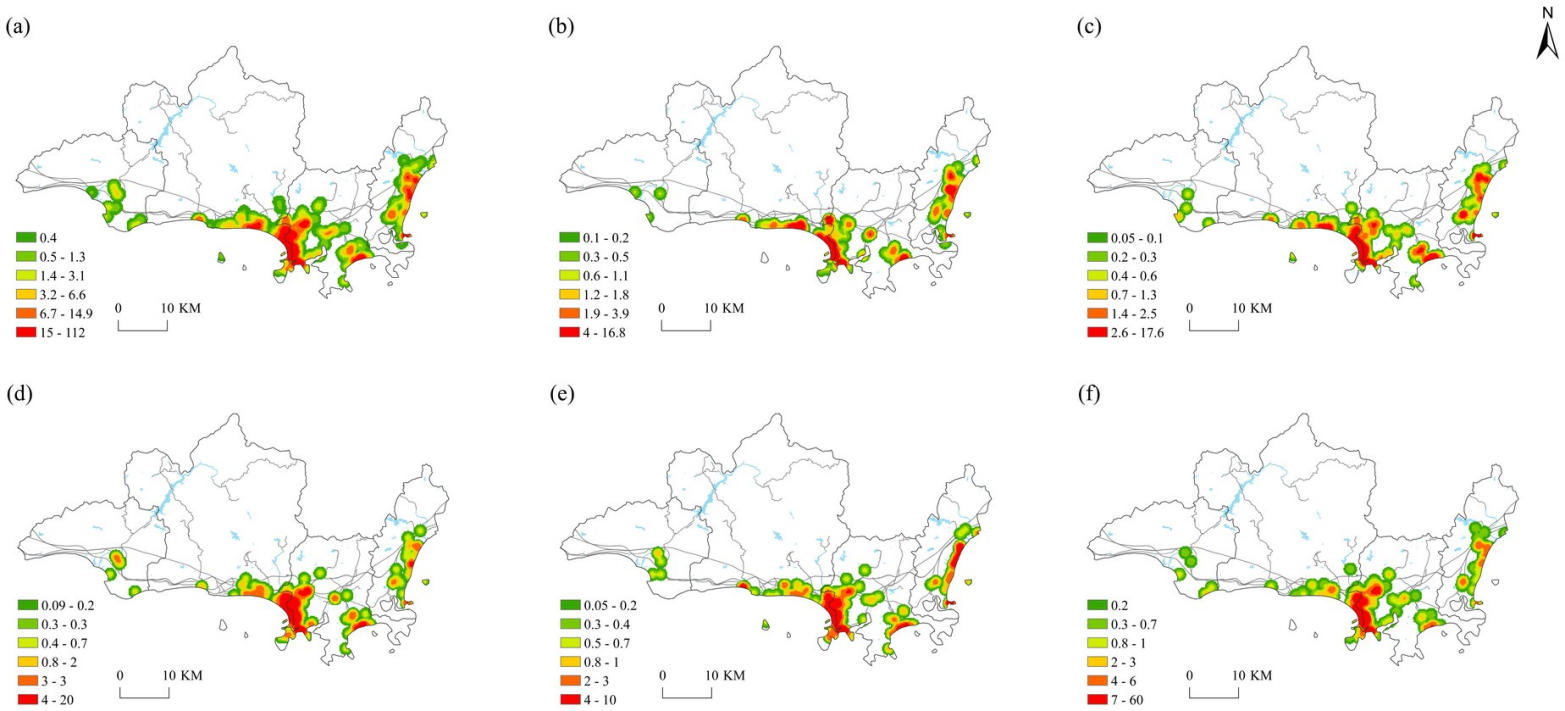
Spatial distribution characteristics of the foodservice industry.

The kernel density map of the overall distribution of restaurants in Sanya (Fig 3) indicates that it is characterized by extension along the southern coastline with little dispersion and multiple clusters. The most dense clustering appears at the junction of the Tianya and Jiyang districts and in the southeast bay of the Jiyang district. The medium density area is distributed in the south of the Tianya district, the east of the Haitang district, and along the coastline of the city’s main road. Low-density areas are identified in the southeast of the Yazhou district, the south of the Tianya district and the middle of the Jiyang district.

Additionally, the foodservice industry in the northern area of Sanya does not exhibit a distinct spatial pattern, but the overall spatial distribution is denser in the south than it is in the north. The restaurants in Tianya are mainly concentrated in the Sanya Bay Tianyahaijiao Scenic Area, the 10,000-person seafood square and around the Jinjiiling Scenic Area. Jiyang has a medium-high concentration of restaurants in the Sanya Municipal Government, Phoenix Ridge Park, Baihua Valley Commercial Center and Yalong Bay Outlet areas. Haitang has a number of high-density concentration areas around Houhai Village and the hotel group along Haitang Bay, and each concentration features concentric circles with decreasing density (Fig 3a).

### Distribution characteristics of various types of restaurants

Dining is one of the ways people experience different cultures, so its spatial patterns can reflect those of different consumer groups and restaurants. The foodservice industry can be divided into five categories [45].

The diversity of food and beverage enterprises in Sanya is related to its unique characteristics. Tianya has the largest number of seafood restaurants, followed by Jiyang, Haitang, and Yazhou. These restaurants are mainly distributed in Sanya Bay, Dadonghai, Yalong Bay, and the Haitang Bay coastline along and along the city’s main road. Tianya, Jiyang, and Haitang have several large high-density agglomerations, such as the Haiyu West Line along the Tianya Cape tour area, the Coconut Dream Promenade, Sanya River, New Town Road, Sanya Bay Road, Jiyang Dadonghai, Yuya Road, the Baihua Valley commercial center and the outlet along the coastline of Yalong Bay, which form a contiguous medium- and high-density cluster around the hotels near Haitang Bay (Fig 3b).

The spatial pattern of Hainan cuisine is similar to the seafood. There is a large high-density agglomeration that is widely distributed. The Tianya, Jiyang, and Begonia districts form a continuous agglomeration in Yazhou around the Nanshan cultural tourism area. The Sanya Bay coast forms three circular high-density agglomerations from the coastal zone to the inland expansion that sequentially decrease. The Hundred Flowers Valley and Tropical Forest Park form a double-core high-density agglomeration, and the Jiyang Haitang Bay coastline and Haiyu East Line form an irregular strip-shaped area (Fig 3c).

Foreign restaurants form high-density agglomerations in all four administrative districts, especially in the Tianya and Jiyang districts in areas such as Dadonghai, the Fenghuangling Haiyinshanmeng Scenic Area, and the northern section of Yingbin Road. A large, contiguous high-density agglomeration is formed along Lijiegou, Baihua Valley, Commodity Street and the Atlantis Resort along Haitang Bay, the Haitang Bay Duty Free Plaza and the Mangrove Resort Hotel. There is a small, circular high-density agglomeration on Yazhou Road in Yazhou (Fig 3d).

The high-density distributions of international restaurants are similar to the overall spatial pattern of the restaurant industry in Sanya, with four obvious concentrations at the junction of the Tianya and Jiyang districts that form a strip along the coastline of Yalong Bay and Haitang near the Sanya International Duty Free City and the hotel complex along Haitang Bay. The high-density distributions in Yazhou and Tianya districts are fewer, and their shape is more compact with a concentric oval structure (Fig 3e).

Casual dining restaurants are the most numerous and varied among all types of restaurants in Sanya, but there are only a few high-density concentrations that form a large agglomeration at the junction of the Jiyang and Tianya districts that is mainly distributed in the First Market, New Wind Street, Pearl Square and near Commodity Street. Small high-density concentrations appear near the hotels along Yalong Bay and Haitang Bay. Small concentric oval-shaped concentrations appear in the middle of Yalong Bay and Begonia Bay, while the rest of the doughnut-shaped low-density areas appear in the southeast of Yazhou, the south of Tianya, and the middle of Jiyang (Fig 3f).

### Spatial distribution characteristics of the foodservice industry in Sanya

The map divides the study area into statistical cells that form a grid and calculates the means of the four types of ratings in each grid to characterize their spatial distribution. We used the natural discontinuity method in ArcGIS to typify categorize the overall development of the foodservice industry into five levels from high to low, as follows: A+ (4.43–4.8 points), A (4.2–4.43 points), B (4–4.2points), C (3.75–4 points) and C− (3.4–3.75 points).

### Spatial variation in the overall eWOM score

**Fig 4:**
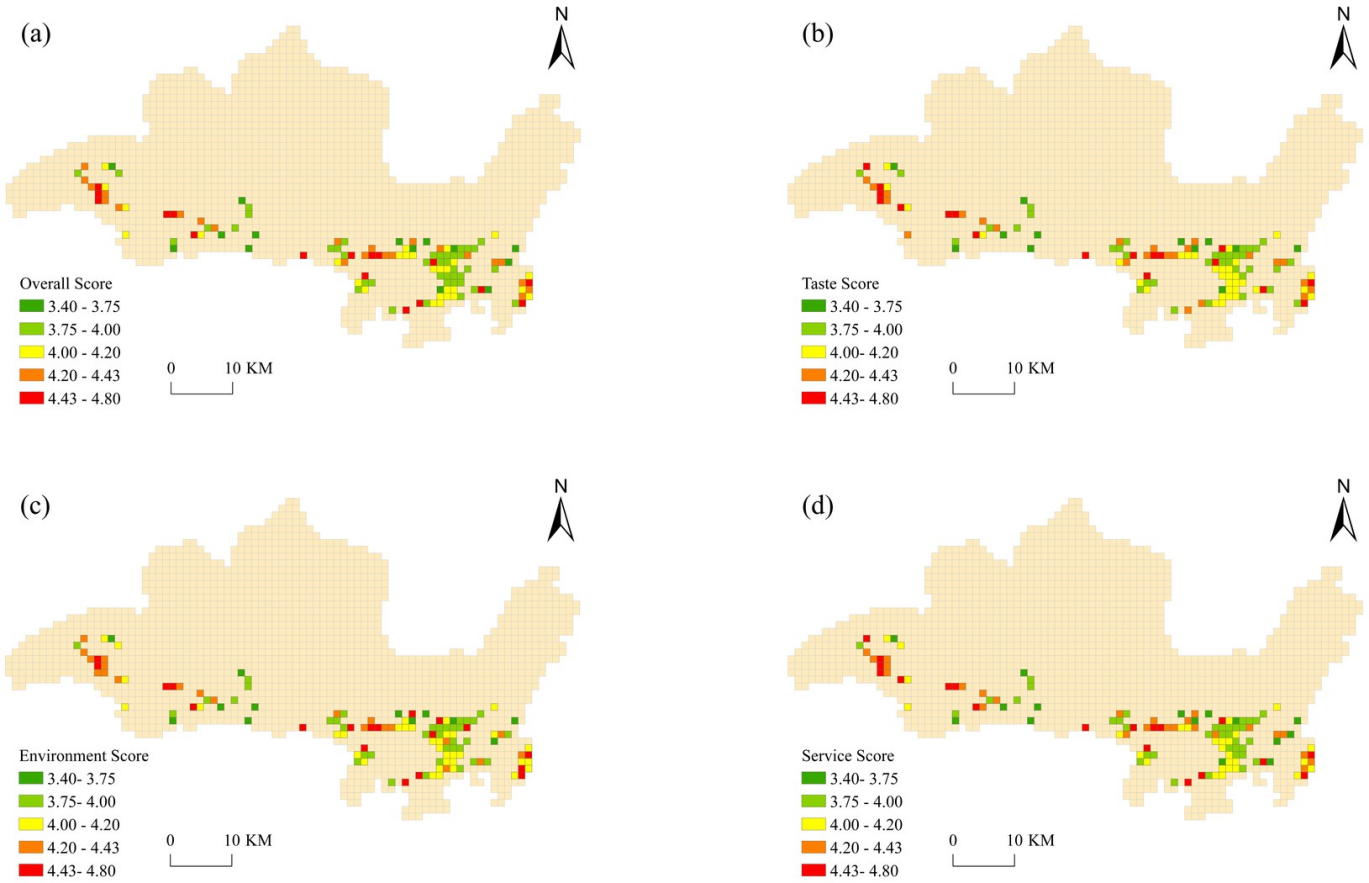
Spatial pattern of electronic word-of-mouth (eWOM) in the foodservice industry in Sanya.

As can be seen in Fig 4, Type A (i.e., A+ and A-grade) grids are distributed in clusters and strips, while Type B (i.e., B-grade) and Type C (i.e., C- and C-grade) grids are clustered in Jiyang and in the southwest area of Tianya. This shows that the scores in the eastern and western areas of the city are generally better than those in the central area of the city. In terms of quantity, there are more Type C grids than there are Type A and Type B grids, thus indicating that the level of eWOM in Sanya is low, with 67% of the scores falling below 4.2. There are differences in visitor satisfaction in the four major administrative regions, but there is no obvious pattern in those differences.

Haitang is generally balanced, with Type A grids found mainly in the coastal tourism development axis and a few Type C grids around the ecological corridors of the southwestern and northern mountain ranges. This indicates that the best e-ratings are found in Haitang. Jiyang has more Type B and Type C grids, and the overall scores of both the central and the coastal tourism areas are low, probably because there are more casual dining restaurants around the scenic spots and the overall score varies. The ratings in Yazhou are similar to those in Haitang, where most grids are Type A grids and thus have good environmental protection and a selection of upscale restaurants.

### Spatial variation across categories

To ensure that the level of eWOM is comparable across dimensions, the same classification criteria as described above were used to categorize taste, environment, and service scores. The spatial pattern of eWOM in the foodservice industry can be revealed by observing and comparing the visitor satisfaction grid maps for each indicator. The spatial distribution of the taste, environment, and service ratings in Sanya is generally consistent while being locally concentrated and widely dispersed, with each type of grid found throughout the study area.

The distribution of the four types of satisfaction vary across the four major administrative districts, among which Type A grids in Haitang are concentrated along the coast of Haitang Bay, while Type B and Type C grids are distributed in the area around the regional administrative center and the central business district. A small number of Type A grids are concentrated near the administrative center and the First Mmarket near the boundary between the Jiyang and Tianya districts, and a large number of Type B and C grids are clustered in the southeastern area of Jiyang. In Yazhou, Type A and B grids are distributed in a cluster around the administrative center, and Type C grids are scattered throughout the study area.

There are 44 Type A grids and 51 Type C grids, and their taste and environment scores are comparable. This shows that there are large differences in restaurant quality in Sanya, which is the main reason for the low ratings of some restaurants. Therefore, restaurants need to pay attention to their service quality as well as the training and management of their employees while formulating strict rules to standardize their services. Only a small number of regions show differences in the spatial distribution of the three types of satisfaction. For example, A and B taste ratings are comparable along the coast of Haitang Bay, but there is also a C grid, while A+ and B environmental ratings are common with no Type C grid, which is also the case for service ratings.

## Section 5: Underlying mechanisms in the spatial distribution of eWOM in the foodservice industry in Sanya

### Factor selection and description

In this study, seven indicators, including population, price, market activity, food quality, transportation conditions, commercial development, and public services are selected to analyze the influencing factors in the spatial distribution of eWOM in the foodservice industry in Sanya using the GWR model.

The selected factors can be described as follows. (1) *Population* [16], was measured as population density. Areas with high population density will have a greater demand for restaurants, but excessive tourism will lead to queuing, a noisy dining environment, damage to the environment and other problems. (2) *Price* [46], was measured as per capita consumption. The per capita food and beverage consumption is measured by a variety of factors and reflects the overall level of service, but excessive consumption will negatively affect tourists’ dining experience. (3) *Market popularity* [47], was measured as the number of online reviews. The market popularity of a restaurant affects tourists’ choices and purchases, and it is the key to the success of restaurant development. (4) *Restaurant rating* [23], was measured as the restaurants’ star rating. It represents their food quality, dining environment and service quality to a certain extent, are closely related to the level of foodservice eWOM. (5) *Transportation conditions* [48], was measured as the number of public transportation stops within 1500 m of the restaurant. Good transportation conditions can improve the convenience of visit the restaurant. (6) *Commercial development* [49], was measured as the number of shopping malls and entertainment venues within 500 m of the restaurant. The flow of people and presence of shopping malls and entertainment venues attracts restaurants and brings prosperity to the tourism industry. (7) *Public services* [50], was measured as the number of hotels, colleges and universities, hospitals, governmental units, and A-grade scenic spots within 1000 m of the restaurant. Public service institutions and facilities such as schools, hospitals, government units and other nearby large number of staff have a high demand for food and beverage services, attracting a large number of high-quality food and beverage enterprises in the surrounding area to focus on development.

To reduce the effects of heteroskedasticity and data outliers, the variables were standardized using the extreme value treatment method.

### OLS regression results

The regression model was constructed by taking overall score of restaurant industry as the explanatory variables and population, price, market popularity, restaurant rating, transportation conditions, commercial development, and public services as the explanatory variables. First, OLS linear regression was conducted to test the direction, effect and significance level of the explanatory variables on the explanatory variables, and the results are shown in Table 1. The results can be summarized as follows. (1) Among the explanatory variables, only public services did not reach the required significance level, while the rest of the variables passed the test of statistical significance. This may be due to the indicators selected in this paper not being fully representative, which may have some influence on the results. (2) The variance inflation factor (VIF) test shows that the VIF of each driver is less than 7.5, which again indicates that the model variables are reasonable and that there is no redundancy or multicollinearity in the variables.

**Table 1:** OLS model test results.

Variable	Coefficient	Standard deviation	T value	P value	VIF
Intercept	2.5260	0.0211	119.5792	0.0000***	——
X1	−0.0282	0.0145	−1.9460	0.0518*	3.1616
X2	0.1458	0.0404	3.6086	0.0003***	1.1665
X3	0.1888	0.0421	4.4897	0.0000***	1.0798
X4	2.2231	0.0204	108.9806	0.0000***	1.4355
X5	0.1213	0.0154	7.8699	0.0000***	2.1956
X6	0.0857	0.0213	4.0202	0.0000***	4.0144
X7	0.0322	0.0230	1.3985	0.1621	6.2292
OLS OLS diagnosis	Joint F value	Jarque–Bera test	K(BP)test	Joint chi-square	
	0.000***	30.8829	19.2397	16648,0.0000***	

Note:

***, **, and * indicate significance at the 0.01, 0.05 and 0.1 levels, respectively.

### Spatial heterogeneity of influencing factors based on the GWR model

Considering that the OLS linear regression model only focuses on broad regression coefficients, this paper further analyzes the specific effects of each factor by constructing a GWR model and conducting a geographically weighted regression analysis using ArcGIS software. When running the GWR model, the parameters are set using the latitude and longitude of each hotel as the geographic coordinates, the kernel type set as the fixed Gaussian function, the Golden Section search used to select the bandwidth, and AICc as the bandwidth selection criterion. According to Brunsdon et al., [44] the GWR model is effective if it produces an AICc value of three or more less than the OLS regression estimation [51]. The results show that the AICc value of the GWR model is −2766.1516 compared to that of the OLS model of −2730.6402, which reflects its suitability.

The GWR model results show the specific coefficients for each spatial unit in Sanya. Table 2 shows the descriptive statistics for each quintile of coefficients estimations. From the maximum and minimum values, it can be seen that the spatial variation in the explanatory variables varies greatly, and the restaurant rating and market popularity have a significantly positive impact on the eWOM level of the foodservice industry in Sanya. The coefficients on population density and price are both positive and negative, thus indicating that their effects on eWOM are mixed. The averages of price, restaurant rating, market popularity, transportation conditions, and commercial development are positive, and that of population density is negative.

**Table 2:** GWR model results.

Variable	Minimum	Lower quartile	Median	Upper quartile	Maximum	Mean
X1	−0.7880	−0.0114	−0.0045	−0.0025	0.0009	−0.0179
X2	−0.4950	0.1568	0.1666	0.1703	0.1828	0.1487
X3	0.0519	0.1955	0.2003	0.2066	2.6769	0.2054
X4	1.8684	2.2473	2.2796	2.2818	2.2865	2.2181
X5	0.0980	0.1000	0.1005	0.1379	0.4302	0.1290
X6	0.4379	−0.023	0.0491	0.0736	0.5578	0.0771

**Fig 5:**
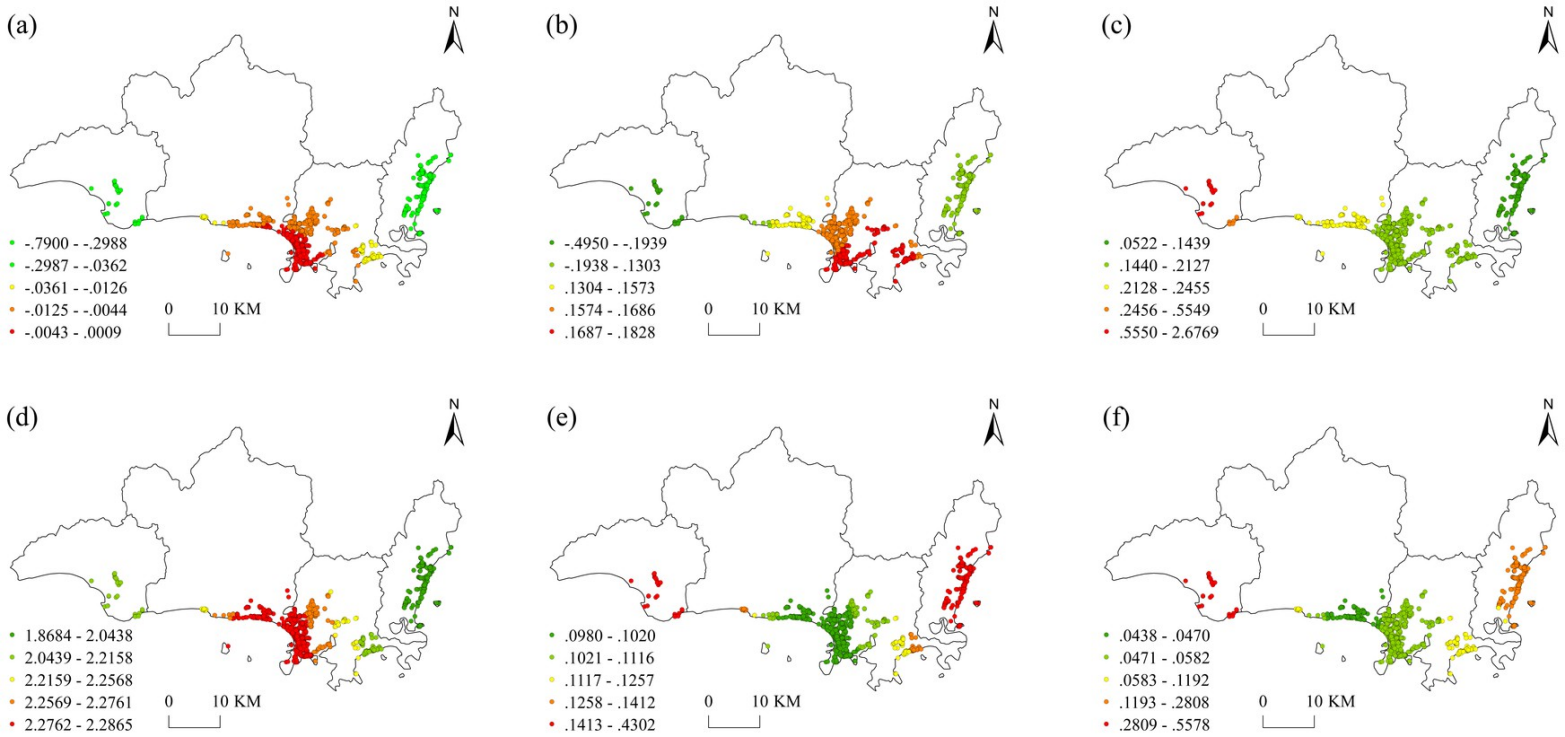
Spatial distribution of regression coefficients using the GWR model.

The factor coefficients were visualized using the Jenks natural break method in ArcGIS software to further portray the spatial differentiation of the regression coefficients on the influencing factors. The spatial distribution of the regression coefficients on the factors shown in Fig 5 indicates that there are significant spatial differences, which reflects the spatial heterogeneity of different impact factors on the level of eWOM in the foodservice industry in Sanya.

Population has the smallest influence among the factors and is negative. The spatial heterogeneity of the regression coefficients are shown in Fig 5a. It can be seen from the figure that the distribution pattern is characterized by being high in the middle and low at both sides of the study area. The coefficients range from −0.7880 to 0.0009, and the high values are clustered in the administrative center, Dadonghai coast, and southeast coast of Sanya Bay as well as the areas with higher population density. Negative values are mainly distributed in Yacheng, the Nanshan Cultural Tourism Zone, Yalong Bay and Haitang Bay coast, which indicates that population density has a significant inhibitory effect on visitor ratings in these areas. The reason for this finding may be that a large number of restaurants are clustered near the high-end hotels on the coast of Yalong Bay and Haitang Bay, whose patrons have high dining standards. The service issues related to high population density will negatively affect restaurants’ ratings, which will in turn affect their eWOM.

The influence of price on the spatial differentiation of eWOM in the foodservice industry in Sanya is not significant. From Fig 5b, it can be seen that price has both positive and negative impacts on eWOM. The spatial distribution of the coefficients increases from the eastern area of the Haitang district and the western area of the Yazhou district to the Jiyang district, and the coefficients range from −0.4950 to 0.1828. The high values are concentrated in Jiyang, the Tianya coastline belt and other areas along the coastal tourism development axis and in the region where the potential for tourism development is significant. Increases in price are conducive to upgrading the quality of the foodservice industry. The low values are found in the Yalong Bay coast and the Yazhou district because the potential for further price increases is limited in those areas. There are extensive ecological protections in Yazhou and thus its potential for restaurant and tourism construction is limited.

Market popularity is the second major influencing factor of spatial differences in the eWOM of the foodservice industry in Sanya. As can be seen in Fig 5c, the eWOM of the foodservice industry in Sanya has significant positive sensitivity to price. Its spatial distribution is varied and increases from the eastern area of the Haitang district to the Yazhou district, with coefficients ranging from 0.0519 to 2.6769. The low coefficients are mainly clustered near the hotel cluster along Haitang Bay in Haitang, the administrative center of Jiyang, Yalong Bay, Dandonghai, and the tourist resort area along Sanya Bay, which are as the tourist hotspots in Sanya. Given that the level of market popularity is already high, additional publicity will not have a significant impact on the growth of its foodservice industry. Yazhou is not a major tourist resort area in Sanya and therefore struggles to attract restaurants.

Restaurant rating has the most significant positive influence on the spatial variation in eWOM in the foodservice industry in Sanya, with coefficients ranging from 1.8684 to 2.2865. As can be seen in Fig 5d, its spatial distribution pattern of its coefficients is high in the middle and low on both sides, with a peak near the boundary line of the Tianya and Jiyang districts. Restaurants’ star grade generally represents the quality of their food, dining environment and service quality, and the ratings are mixed in administrative centers near the boundary line of the Tianya and Jiyang districts as well as the Sanya Bay tourism hotspots such as Tianya and Jiyang. Tourists are generally more satisfied with high-end restaurants, so popularity has a significant impact on restaurants’ ratings. Yazhou is far from the city center and not a popular tourist area, so there are only few restaurants with high ratings. Begonia Bay coast has many high-end hotel clusters, where the food quality and ratings higher, which suggests that further increasing ratings in this area will have less of an impact than it will in the central area of Sanya.

The influence of transportation conditions on the spatial heterogeneity of eWOM in the foodservice industry in Sanya is low. From Fig 5e, it can be seen that its influence is positive and characterized by a spatial pattern that is high in the middle and low on both sides, with coefficients ranging from 0.0980 to 0.4302. The areas with high coefficients are clustered in the Jiao Tou Nose, Yazhou Bay Science and Technology City, Damao Town, and the northern area of Haitang Bay. Because these areas are far from the city center, the impact of public transportation on tourists’ satisfaction will be higher. The government may therefore consider further improving the availability of public transportation in these areas in the future. The areas with low coefficients mainly appear in the Tianya Haijiao Scenic Area, Sanya Bay, Dadonghai, the Jiyang Administrative Center, and around the highways. These areas have dense roads and good traffic accessibility, so the sensitivity of tourist satisfaction to this factor is low. In the future, strengthening the interconnection between public transportation and restaurants, building transportation networks and improving the accessibility of restaurants will attract more tourists and promote high-quality development in the foodservice industry.

The commercial development has a small impact on eWOM in the foodservice industry in Sanya. From Fig 5f, it can be seen that this factor is positively correlated with the commercial development, with coefficients that range from 0.0980 to 0.7020. The high coefficients appear in the Nanshan Cultural Tourism Zone and the Port Gate in Yazhou, which are not popular tourist areas with few restaurants, where an increase in commercial activity can further attract tourists. The tourist resorts in the Sanya Bay coast, Jiyang Administrative Center, Dadonghai Tourism Area, Yalong Bay, Haitang Bay, are mature and have extensive tourism development, so their sensitivity to business activity is low and will not significantly impact high-quality development in the region.

**Fig 6:**
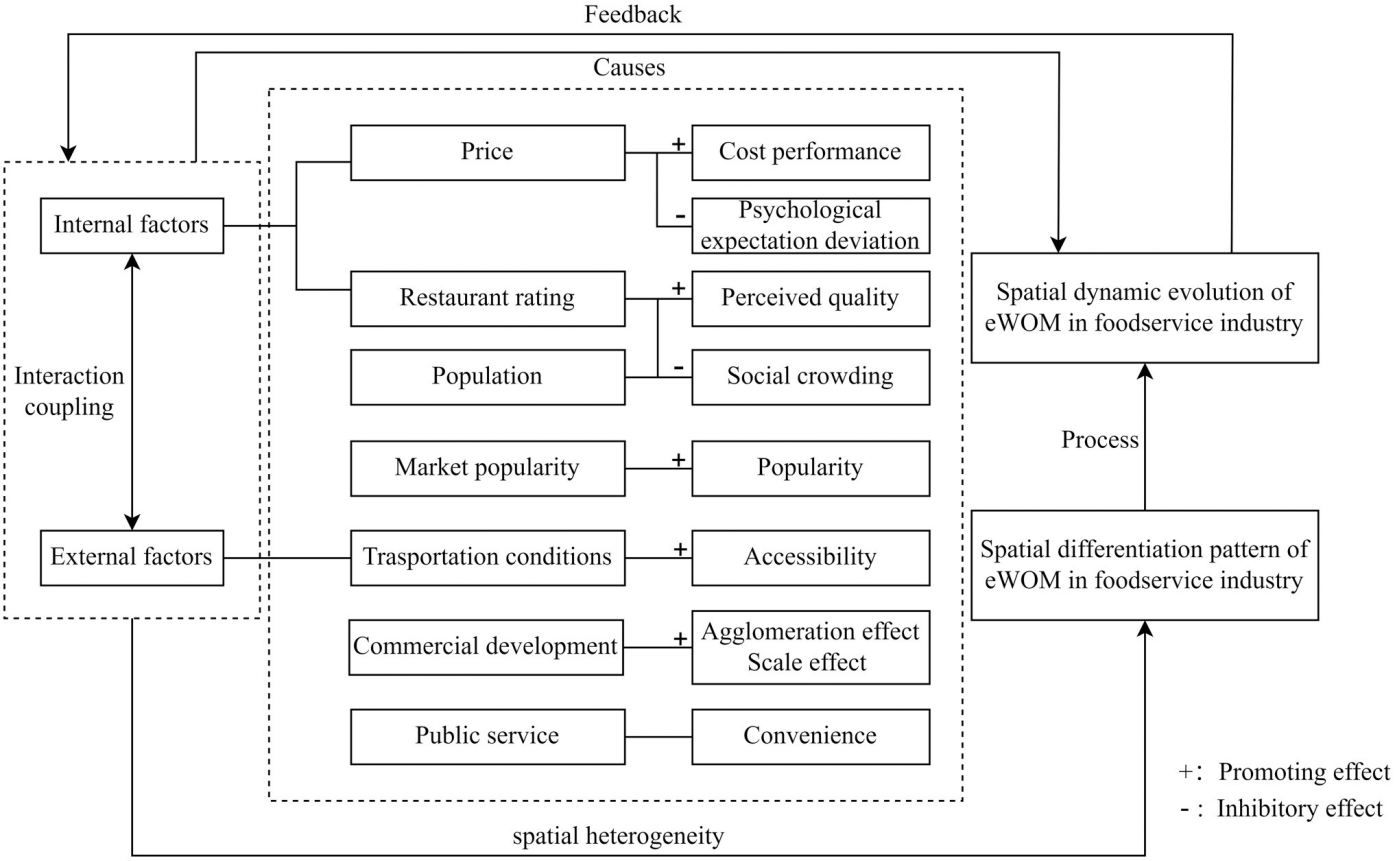
Underlying mechanisms of eWOM in the foodservice industry.

Taking the above analysis, the underlying mechanisms of eWOM in the foodservice industry in Sanya are summarized (Fig 6). Specifically, the interaction and coupling of internal factors and external factors have laid the foundation for the spatial pattern change of eWOM in the foodservice industry. The evolution process of spatial differentiation pattern determines the spatial dynamic evolution characteristics, and the spatial dynamic evolution is fed back to regional policies and main demand. The above processes have formed the underlying mechanism of eWOM in the foodservice industry in Sanya.

## Section 6: Discussion and conclusion

The main conclusions are as follows. The foodservice industry in Sanya is characterized by extension along the southern coastline with little dispersion and multiple agglomerations. The overall distribution is similar and mainly relies on the coast and the city’s main roads. Seafood restaurants, Hainan cuisine, local food high-density are more dispersed, whereas international restaurants and casual dining are more concentrated. In Sanya, the level of eWOM in the foodservice industry is low, as 67% of the ratings were 4.2 points or below. The distribution pattern is generally consistent throughout the region. Ratings in the eastern and western districts are generally better than those in the central district. The eWOM of the foodservice industry in Sanya is influenced by both internal and external factors. Market popularity, restaurant rating, transportation conditions, and commercial development have a positive influence on eWOM in the foodservice industry. Population and price have both positive and negative influences on it, while the effect of public services is not significant.

According to this study, The foodservice industry in Sanya extends along the southern coastline and the overall distribution is similar and mainly relies on the coast and the city’s main roads. This finding is similar with earlier studies in the field. Xu et al. argued that high-level business districts, with their established facilities and transportation accessibility, attract a variety of high-quality restaurants, thus improving their eWOM [4].

The overall eWOM score of the foodservice industry is low. The reason may lie in the fact that there are many types of food and beverage around the key tourism development areas and coastal tourism areas, and that there are more affordable snack stores such as Snack Street, with varying levels of overall standards, which need to be focused on and improved, and the development of high-grade food and beverage outlets.

The results show that price, market popularity, restaurant ratings, transportation conditions, and commercial development all positively influence eWOM in the foodservice industry. The availability public services does not have a significant influence, which is consistent with previous findings [46–50]. Population has a negative influence, as high concentrations of tourists may lead to problems such as long wait times, noisy dining environments, and traffic congestion [49].

In addition to these influencing factors, Sanya’s unique cultural characteristics will also affect tourists’ ratings of restaurants, thus affecting their eWOM. For example, seafood is the largest number of restaurant types in Sanya City, which is related to the characteristics of Sanya’s ‘marine culture’, and the freshness and deliciousness of the seafood will promote the tourists to give higher ratings, but the situation of rip-offs and overpricing of seafood in some seafood markets will also affect the reputation of the foodservice industry in Sanya City. However, some seafood market rip-offs and overpriced seafood will also affect the reputation of the foodservice industry in Sanya [51, 52]. In addition, the people of Sanya have lighter dietary tastes, part of the foodservice such as hot pot will adjust the taste, which will give higher ratings for the light diet of tourists, but part of Sichuan Province, Chongqing and other heavier tastes of tourists will also give a poor evaluation, affecting the reputation of the foodservice industry in Sanya [53]. Finally, with Hainan’s distinctive culture, such as the Li-Miao culture of the restaurants is relatively small, operators can make more use of and excavation of the local characteristics of the culture, to give tourists novelty characteristics of the experience, in order to improve the Sanya foodservice industry’s eWOM [54, 55].

### Implications

The foodservice industry is not only an important industry for urban development, but also an important element of urban tourism. In particular, the rapid development of information technology has led to the widespread popularization of online platforms such as Volkswagen Dianping and Meituan. Review data have become an important reference for users in making purchase decisions [6, 7]. Therefore, exploring eWOM in the foodservice industry in this context is of great significance.

### Theoretical contributions

At present, studies on eWOM in the foodservice industry are mainly in the fields of management and psychology. The main objective of these studies is to explore its relationship with consumers’ purchase decisions and satisfaction [33, 35], while its spatial distribution patterns and influencing factors have been less studied. In this study, kernel density analysis, grid analysis and the GWR model were used to analyze the distribution characteristics and influencing factors of eWOM in the foodservice industry in Sanya. Our results extend those of the existing literature. In this study, Sanya, a tourist city, was selected as a case object to explore the spatial distribution characteristics and influencing factors of eWOM in the foodservice industry. Previous studies have focused more on the foodservice industry in economically developed megacities such as London and Beijing [15, 27], but there is little research on small and medium-sized cities. Sanya is an internationally recognized tourist city with a rich and diverse foodservice industry. Taking Sanya as an example not only enriches the existing research, but also provides a theoretical and practical reference for the improvement of eWOM in the foodservice industry in comparable cities.

### Practical contributions

Spatial analysis methods were used to explore the spatial distribution characteristics and influencing factors of the eWOM of the foodservice industry in Sanya. The results can be referenced by both governments and practitioners to guide future development. Due to differences in population, price, market popularity, restaurant rating, transportation conditions, and commercial development, it is found that different regions have significant spatial imbalances in foodservice industry eWOM. This paper argues that, to further improve the level of eWOM in the foodservice industry, narrow the gap between regional eWOM levels, and promote high-quality and balanced development in the foodservice industry, it is necessary to adhere to the principles of localization, zoning, and classification as well as to formulate practical and effective development strategies. In the Tianya and Jiyang districts, on the one hand, the areas with low values should adjust their prices to improve the level of service quality. On the other hand, the government should construct new infrastructure and offer forms of policy support to develop more mid-range and high-end restaurants. In Haitang and Yazhou, the high-value areas should make full use of their advantages to drive high-quality development in neighboring areas.

### Limitations and suggestions for future research

OTA scoring data are only one perspective of eWOM in the foodservice industry, and thus the results may not be generalizable to other areas. In the future, it is necessary to further expand this study using alternative measurements. Only a single year is studied, so it will be valuable for future research to broaden this study to include additional time periods. Foodservice industry eWOM is subject to the influence of many factors. However, in this study, only seven factors are explored, which may be insufficient. Therefore, other factors can be included to build a more comprehensive index system.

## Supporting information

S1 Data Score. (XLSX)

S2 Data Location of the foodservice industry in Sanya. (XLSX)

S3 Data Factors. (XLSX)

S4 Data GWR coefficient. (XLSX)
